# Multi-task learning for predicting pulmonary nodule growth and follow-up volume

**DOI:** 10.3389/fonc.2026.1677521

**Published:** 2026-02-03

**Authors:** Wenjuan Zhao, Yang Chen, Yuangzhong Xie, Shengdong Nie, Baosan Han, Yue Jiang, Xiujuan Li

**Affiliations:** 1 School of Health Science and Engineering, University of Shanghai for Science and Technology, Shanghai, China; 2 Medical Imaging Center, The Affiliated Taian City Central Hospital of Qingdao University, Taian, Shandong, China; 3 Department of Breast Surgery, Xinhua Hospital Affiliated of Shanghai Jiao Tong University School of Medicine, Shanghai, China; 4 Department of Thyroid and Breast Surgery, The Affiliated Shunde Hospital of Jinan University, Foshan, Guangdong, China

**Keywords:** deep learning, follow-up, growth prediction, multi-task learning, pulmonary nodule

## Abstract

**Hypothesis:**

The primary objective of this study is to develop an end-to-end deep learning framework based on multi-task learning to predict pulmonary nodule growth by jointly modeling nodule segmentation and visual follow-up image synthesis. By decoupling nodule growth into deformation and texture evolution, the model aims to enhance predictive accuracy and clinical applicability through improved regional focus and deep supervision strategies.

**Methods:**

We present MT-NoGNet, a dual-task network for pulmonary nodule growth prediction via simultaneous deformation-texture modeling. The framework employs a shared encoder with two decoders: a spatial transformer for volume change estimation and a texture predictor with adaptive normalization. A cross-task attention mechanism enforces consistency between morphological expansion and internal density evolution.

**Results:**

Evaluated on longitudinal CT scans from 246 patients at Shanghai Chest Hospital, the framework achieved that the predicted peak signal to noise ratio (PSNR) was 44.30, structural similarity index (SSIM) was 0.7776, and dice similarity coefficient (DSC) was 0.7823.

**Conclusions:**

This study establishes that multi-task learning model of deformation-texture features significantly enhances pulmonary nodule growth prediction accuracy while providing radiologists with interpretable visualizations of progression patterns, demonstrating substantial potential for optimizing clinical surveillance protocols.

## 1 Introduction

Pulmonary nodules are common indications of early-stage lung cancer, but not all develop into lung cancer. Therefore, it is necessary to carry out different classification management strategies for nodules with different risk levels. Early detection, diagnosis, and treatment of high-risk nodules would effectively enhance lung cancer survival. Computed tomography (CT), with its superior imaging capabilities—submillimeter nodule resolution (0.3-0.5 mm) and low-dose protocols—is the primary method for pulmonary nodule screening and follow-up (1). Radiologists predominantly rely on CT images for lung lesion identification. However, the morphological diversity and complexity of pulmonary nodules pose a significant challenge in identifying and assessing the malignancy of some small, ambiguous nodules (2). The clinical criteria for clinically assessing the malignancy of pulmonary nodules is performed visually by observing the diameter changes of nodules on CT images scanned at different times, and follow their progression (3). In the measurement of the diameter of pulmonary nodules, while the three-dimensional evaluation is a more accurate method of diameter measurement (4). it requires the segmentation of nodules from CT images. Manually Segmenting pulmonary nodules pixel by pixel is not only a time-consuming and laborious process, but also sensitive to subjective and objective factors, leading to inaccurate results. As a result, it is challenging to extend this step into the clinical workflow. In certain studies, the measurement of nodule diameter has been integrated into the lung nodule detection model to facilitate the prediction of nodule diameter (5, 6). In addition, it is also possible to establish a lung nodule segmentation model to achieve pixel-by-pixel prediction of nodules, and then determine the diameter of the nodules through morphological calculations (7). However, due to variations in size, texture, and morphology of nodules, even experienced doctors may have different opinions on some difficult-to-diagnose nodules. In order to help radiologists and clinical doctors judge the malignancy of nodules, experts have developed clinical guidelines, such as NCCN (8), Fleischner (9), and Lung-RADS (10), which provide recommendations for managing pulmonary nodules. Nevertheless, for nodules with complex textures, diagnosis still heavily relies on the radiologist’s experience.

The emergence of CAD system has improved the accuracy and efficiency of clinical doctors in screening pulmonary nodules, which has also reduced decision biases caused by clinical experience differences. At present, many CAD studies have proposed methods for detecting (11–18), segmenting (19–24), and distinguishing (25–27) between benign and malignant pulmonary nodules, and have achieved encouraging results. However, due to a lack of available datasets and annotations for research, few studies analyzed and investigated the growth of pulmonary nodules during follow-up (28). For this study, patients must have received at least one follow-up image, and matched and labeled the corresponding nodules based on two scans. P Huang et al. (29) and colleagues analyzed the follow-up problem of pulmonary nodules, but did not model the appearance changes of the nodules. X Rafael-Palou et al. (30) proposed a 3D Siamese network to predict the diameter changes of nodules, but the limitation of this method was also the lack of visual images of nodule changes. Y Li et al. (31) research provided a visual prediction of follow-up images of pulmonary nodules, which was the first study to provide a visual morphological change of pulmonary nodules during growth, providing a good basis for visual assessment of nodule growth prediction. This method requires cascading two deep learning models to achieve prediction, and also requires manual labeling of nodule masking as input, which undoubtedly limits the clinical application of this method.

Although the aforementioned studies represent preliminary advances in predicting pulmonary nodule growth, several critical bottlenecks hinder their widespread clinical adoption. Firstly, a significant disconnect exists between model functionality and the clinical diagnostic pathway. Radiologist assessment of nodule progression is a holistic process that synthesizes morphological changes (size, shape) with internal feature evolution (density, texture). In contrast, prevailing models often focus on a single dimension—predicting only diameter changes (30) or employing cascaded frameworks that process deformation and texture separately (31). This sequential and fragmented modeling approach fails to emulate the synchronous, interactive judgment performed by clinicians, resulting in decision-support information that is one-dimensional and insufficient for comprehensive clinical decision-making. Secondly, the integration of these models into clinical workflows remains suboptimal. Many high-accuracy models (e.g., (31)) still require manually annotated nodule masks as input during inference. This step is impractical in the demanding routine of a radiology department, posing a major obstacle to translational implementation. A truly practical system must be capable of end-to-end processing of raw CT images. Finally, challenges persist in the interpretability and reliability of predictions. Cascaded models inherently risk error propagation, where inaccuracies in the initial deformation prediction directly distort subsequent texture estimation. Furthermore, “black-box” predictions that lack intermediate visualizations make it difficult for radiologists to verify and trust the model’s output.

Multi-task learning aims to improve learning efficiency and predictive performance by learning multiple tasks’ objectives from a shared representation through the complementary and shared information between tasks. In the CAD method for pulmonary nodules, progress has also been made in related research. LH Liu et al. (35) proposed a multi-task learning-based MTMR-Net for analyzing the correlation between pulmonary nodule classification and attribute scores. CL Wang et al. (36) achieved segmentation of multiple anatomical structures, including lung, heart, and clavicle, in chest X-rays using a multi-task learning FCN. BT Wu et al. (37) proposed an end-to-end multi-task CNN model that simultaneously predicts the malignancy degree, attributes, and segmentation mask of pulmonary nodules. WH Liu et al. (38) proposed a multi-task learning model that integrates lung parenchyma segmentation and pulmonary nodule detection, using lung parenchyma segmentation as an attention module in the nodule detection task and improving the detection performance. These studies suggest that multi-task learning can better utilize feature information between tasks, which is expected to improve the generalization performance of models.

Therefore, this study aims to develop an end-to-end deep learning model based on multi-task learning to achieve pulmonary nodule growth prediction. Building upon the hypothesis proposed by Y Li et al. (31) that decouples growth prediction into deformation and texture evolution components, we further posit that deformation prediction primarily focuses on volumetric changes while textural prediction emphasizes feature refinement of nodule patterns. The integration of these complementary tasks is expected to enhance both global and local features, thereby improving growth prediction accuracy.

This decoupled modeling strategy is supported by a growing body of work in medical image analysis. For instance, K Yan et al. (32) demonstrated that integrating lesion morphology with internal texture information improves the detection and classification of abnormalities in large-scale radiological datasets. Similarly, JM Wolterink et al. (33) successfully separated shape and intensity features for coronary calcium scoring, highlighting the benefit of using dedicated network branches to represent structural and textural characteristics. Y Xie et al. (34) further underlined the complementary role of nodule shape and density features in discriminating benign from malignant lesions, which aligns with the proposed architecture—incorporating a deformation pathway to capture spatial expansion and a texture pathway to model internal pattern progression. Together, these studies offer robust empirical support for the multi-task framework, where both specialized branches work in concert to enhance the characterization of nodule growth dynamics.

The contributions of this paper can be summarized as follows: (1) We present a novel multi-task learning framework that jointly performs pulmonary nodule growth prediction and lung nodule segmentation in a single model. To the best of our knowledge, this is the first study that simultaneously achieves these two tasks. (2) To mitigate the inaccuracy in texture prediction caused by incomplete nodular region coverage in deformation predictions, we introduce Tversky loss to enhance nodular region accommodation. (3) For texture prediction, we further propose two distinct deep supervision strategies that optimize network parameters by quantifying the similarity between the final image obtained by the network and the real follow-up image, thereby improving the overall performance of the prediction task.

## 2 Materials and methods

### 2.1 Dataset

To achieve the prediction of pulmonary nodule growth images, this study collected data from 246 patients at Shanghai Chest Hospital. Each patient contained a follow-up nodule that was marked by a doctor, for a total of 246 nodules. These patients had at least two lung CT scans, including a baseline and at least one follow-up. Some patients had multiple follow-up CT images. The image layer thickness ranged from 0.5mm to 1.5mm, and the follow-up time ranged from 8 to 1392 days. As some nodules had multiple follow-up images, this study divided them into pairs in chronological order, such as T0, T1, and T2, corresponding to two follow-up scans. According to this grouping method, the nodules were divided into three sample sets: T0&T1, T0&T2, and T1&T2. Overall, there were 539 sample sets from 246 nodules. The average follow-up time for the samples was 376 days. To ensure a rigorous evaluation and prevent data leakage, we implemented a patient-level data partitioning strategy for all experiments. Specifically, the 246 patients were randomly divided into five mutually exclusive folds. During 5-fold cross-validation, all sample pairs from the patients within one fold were collectively used as the test set, while pairs from patients in the remaining four folds were used for training. This strict patient-level separation guarantees that the model is never exposed to any imaging data from the test patients during training, providing an unbiased estimate of model generalizability.

### 2.2 Preprocessing

To maintain consistent image resolution, the samples were resampled to 1mm^3^ voxels, despite variations in resolution and layer thickness between them. Nodules spanning 3-30mm in diameter were centered and cropped into 48×48×48 pixel volumetric regions of interest (VOIs), sufficiently encompassing all morphological features. All nodular images were adjusted for window width and level to enhance contrast, with adjusted images corresponding to CT values between [-1024, 400], and normalized to between [-1, 1].

### 2.3 Network framework

**Figure 1:**
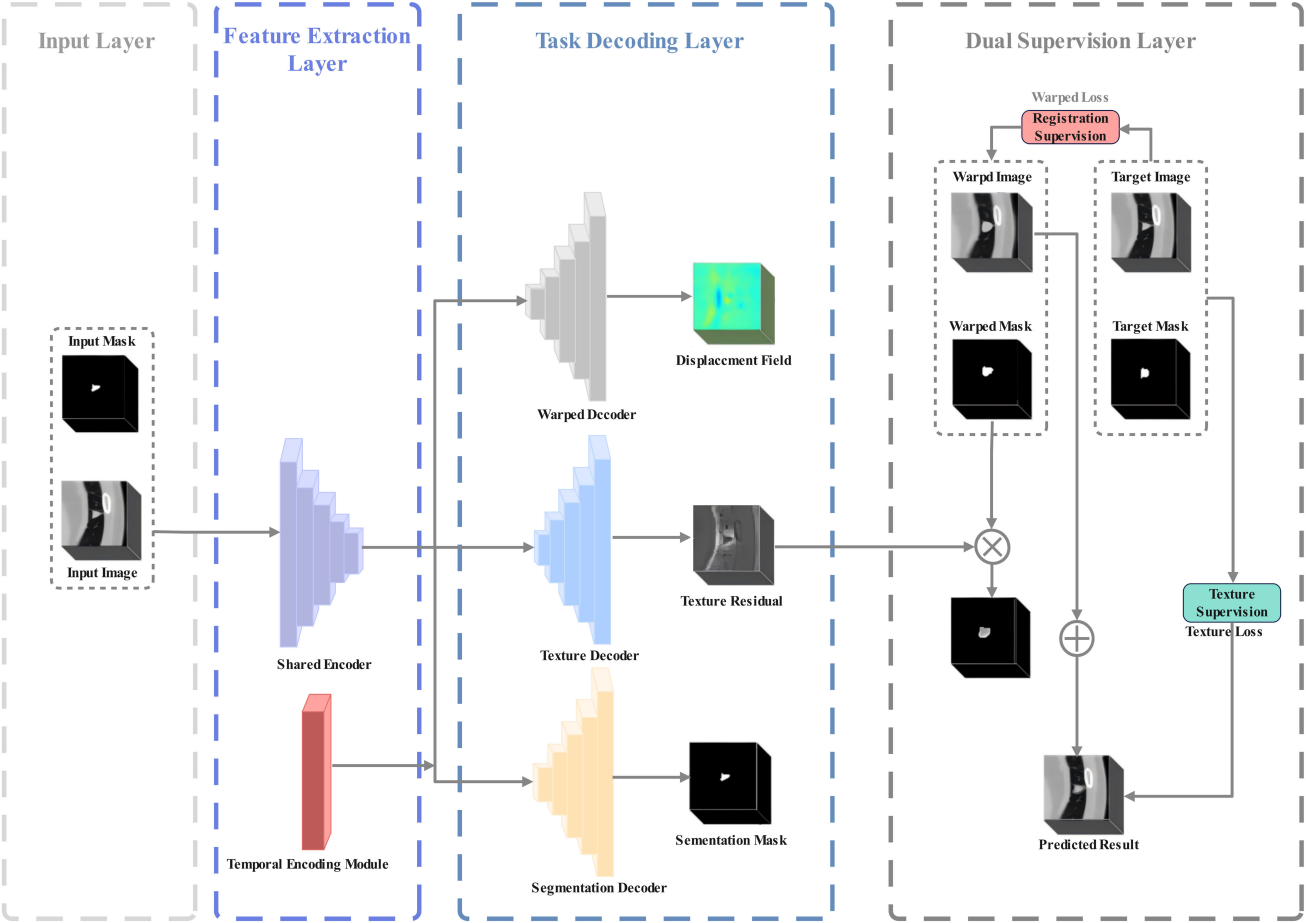
The prediction framework of pulmonary nodule growth based on multi-task learning. Input Layer takes baseline nodule image/mask; Feature Extraction Layer has Shared Encoder (extracts multi-scale 3D features) and Temporal Encoding Module (encodes follow-up time); Task Decoding Layer includes Warped Decoder (outputs displacement field for morphology), Texture Decoder (generates texture residual for density), and Segmentation Decoder (outputs segmentation mask for supervision); Dual Supervision Layer uses Registration/Texture Loss to optimize warped/image/texture predictions against target data.

Our proposed Multi-Task Pulmonary Nodule Growth Prediction Network (MT-NoGNet) employs a dual-branch architecture to simultaneously predict morphological deformation and internal texture evolution of pulmonary nodules, as illustrated in **Figure 1**. The framework operates through three principal components: 1) A shared convolutional encoder that extracts common feature representations from input CT images; 2) Two specialized decoders generating task-specific predictions; and 3) Cross-task constraints that ensure physiological consistency between deformation and texture changes. The network architecture strategically decomposes nodule growth prediction into complementary sub-tasks. The deformation prediction output actively informs the texture prediction process through spatial attention guidance, ensuring that density changes conform to the predicted morphological expansion patterns. This hierarchical interaction mimics clinical observation patterns where radiologists typically assess density changes within the context of lesion morphology. It is important to note that while our model is trained with mask supervision, it eliminates the need for manual mask annotation during clinical inference. Unlike prior cascaded approaches (31), our end-to-end, multi-task framework automatically generates the necessary segmentation internally, thereby significantly improving clinical workflow efficiency.

Specifically, Given a pair of input images {*x_i_,s_i_,t_x_*} and {*y_i_,s_t_,t_y_*}, where *x_i_*​ and *y_i_* represent nodule images at the baseline and follow-up time, respectively, *s_i_* and *s_t_* are the corresponding segmentation mask images, and the follow-up time interval is *t_itv_=t_y_−t_x_*. Inspired by the registration framework proposed by G Balakrishnan et al. (39) and its successful application to pulmonary nodule growth prediction by Y Li et al. (31). This study applies this approach to the volume prediction of nodules.

As described in Equation 1, the deformation prediction task branch learns the displacement field *u* of the nodule volume after a follow-up time interval *t_itv_* by inputting *x_i_* and *t_itv_*. The displacement field *u* is fitted by the deformation prediction function *F_warp_*, where *θ_encoder_* and *θ_warp_* are the parameters of the shared encoder and the deformation prediction task decoder, respectively.

(1) $$u=F_{warp}(x_{i},\theta_{encoder},\theta_{warp},TEM(t_{itv}))$$

As described in Equation 2, *ϕ* is used for the spatial transformation of the nodule, where *ϕ=id+u*, and *id* is the identity function.

(2) ϕ=id+u

After the spatial transformation, the deformation-predicted image *x_warp_* and the corresponding deformation segmentation mask *s_warp_* of the lung nodule are obtained as described in Equations 3 and 4.

(3) $$x_{warp}=\phi \circ x_{i}$$

(4) $$s_{warp}=\phi \circ s_{i}$$

The texture prediction task predicts the residual image between the baseline time *t_x_* and the follow-up time *t_y_* within the region constrained by the deformation segmentation mask *s_warp_*, as described in Equation 5.

(5) $$x_{res}=F_{texture}(x_{i},\theta_{encoder},\theta_{texture},TEM(t_{itv}))$$

As described in Equation 6, the growth prediction image of the nodule can be represented as:

(6) $$\hat{y}=x_{warp}+x_{res}\times s_{warp}$$

where ŷ and s_warp_ are the final predicted nodule follow-up image and its corresponding segmentation mask image, respectively.

### 2.4 Network structure

**Figure 2:**
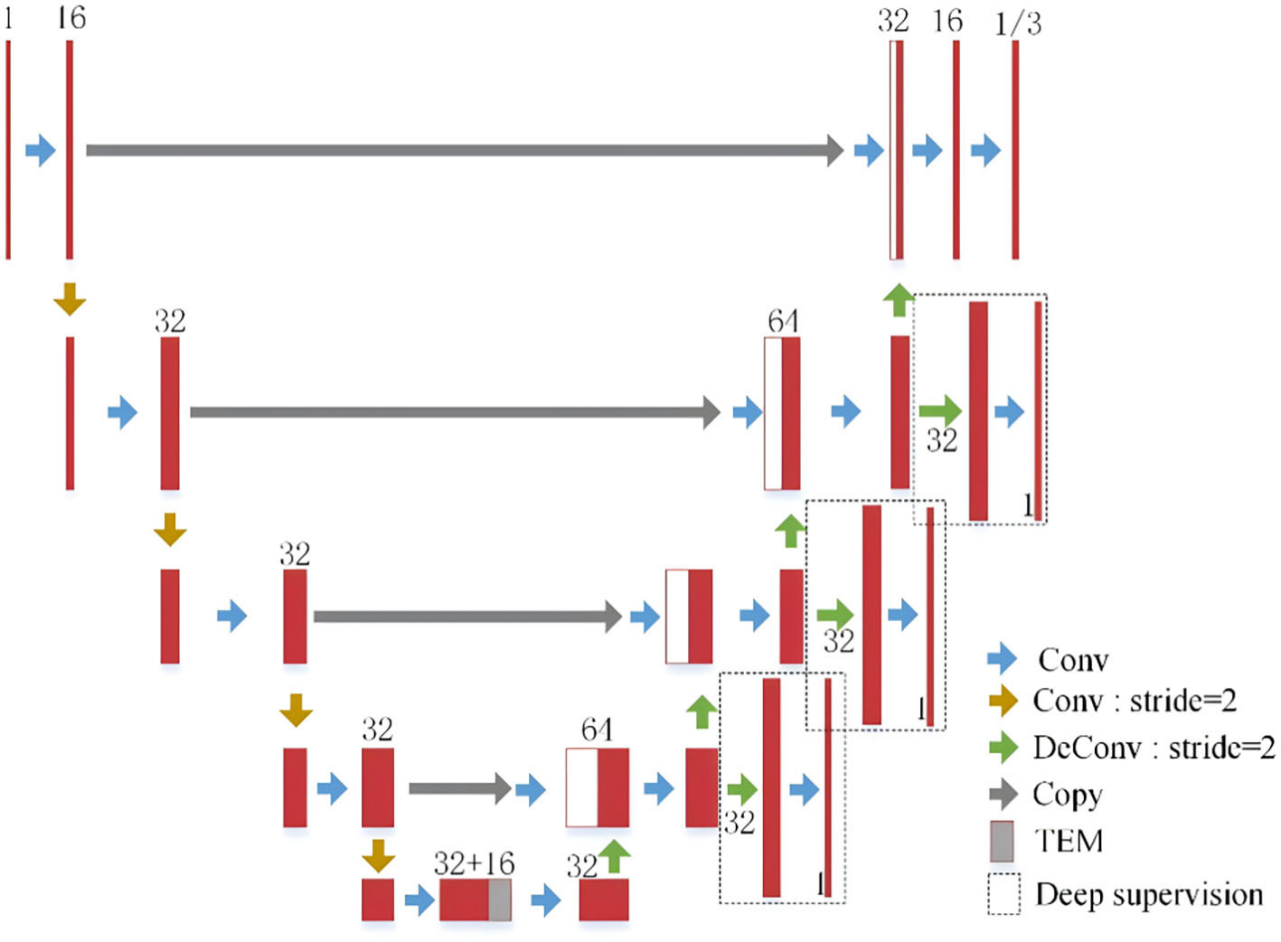
MT-NoGNet network structure. Conv (blue): feature extraction; Conv: stride=2 (yellow): downsampling; DeConv: stride=2 (green): upsampling; Copy (gray): skip connection; TEM (gray block): encodes follow-up time; Deep supervision (white): intermediate supervision. Network downsamples via strided convolutions, fuses time features, uses deep supervision, and upsamples via transposed convolutions + skip connections for final predictions.

As illustrated in **Figure 2**, the architecture of the proposed MT-NoGNet builds upon the 3D U-Net backbone (40), enhanced by a temporal encoding module to enable temporally-aware growth prediction across different timepoints. The shared encoder between the deformation prediction task (estimating spatial displacement fields) and the texture prediction task (modeling intensity variations) facilitates joint learning of nodule characteristics under dual-task supervision. To strengthen multi-task representation capacity, additional convolutional layers are incorporated into the skip connections, facilitating task-specific feature differentiation. Due to the similarity between baseline and follow-up nodule images, standard networks struggle to learn distinct features. Building on GD Zeng et al.’s deep supervision concept (41), we developed a dual-supervision framework to enhance feature learning in the texture prediction decoder. In the early stages of the decoder, this study used the “direct supervision” strategy, which is relative to the “residual supervision” strategy used before. By introducing the direct supervision strategy, the network is encouraged to extract as many nodule features as possible from the encoder part. In the final output of the decoder, this study used residual supervision (as described in Equation 6), which combines the image *x_i_* of the baseline moment to reduce the difficulty of model fitting. The process of direct supervision can be described as follows (Equation 7):

(7) $$\hat{y}=x_{warp}\times (1-s_{warp})+x_{texture}\times s_{warp}$$

In this final integration, *ŷ* represents the predicted follow-up nodule image. This result is synthesized from two core components: the spatially transformed baseline image *x_warp_*, which captures the geometric deformation of the nodule, and the residual image *x_texture_*, which models the intensity and textural evolution within the nodule region. The fusion of these components is governed by the deformed segmentation mask *s_warp_*, which defines the spatial extent of the nodule. This mask-based blending strategy ensures that the predicted texture is applied precisely within the nodule area, while the warped background information is preserved elsewhere.

Specifically, the network takes the baseline nodule image xixi​ with a size of 48×48×48 voxels as input. Owing to its fully convolutional structure, the network is not theoretically constrained by a specific input size during prediction. Downsampling in the shared encoder is achieved through 3D convolutions with a stride of 2, employing a LeakyReLU activation function (negative slope parameter of 0.2) to prevent neuron saturation. In the decoder, transpose convolutions are used for upsampling to restore spatial resolution, ensuring the final output size matches the input.

To condition the model on the follow-up duration, a Temporal Encoding Module (TEM) is integrated to map the scalar time interval *t_itv_* into a high-dimensional feature vector. This allows the network to generate predictions for arbitrary future time points. The follow-up time interval is defined as *t_itv_*=[(*t_y_*-*t_x_*)/30], quantizing time into monthly bins and yielding a prediction range of 1 to 20 months. Follow-up times are truncated at 600 days.

Inspired by the sinusoidal positional encoding and its application in Y Li et al.’s framework (31), the TEM encodes titvtitv using a series of sine and cosine functions with geometrically increasing wavelengths. This provides a unique, continuous representation for each time interval, facilitating the model’s ability to learn temporal dependencies. The encoding for each dimension of the output vector is computed as follows (Equations 8, 9):

(8) $$TEM(t_{itv},2i)=sin(t_{itv}/100^{2i/d_{fm}})$$

(9) $$TEM(t_{itv},2i+1)=cos(t_{itv}/100^{2i/d_{fm}})$$

Here, *i* denotes the dimension index, and *d_fm_* is the total dimensionality of the temporal feature embedding.

### 2.5 Loss function

The shape prediction task requires the volume prediction of the input image $x_{i}$ and segmentation mask $s_{i}$. It is necessary to measure the similarity between the shape-deformed image and the follow-up image, as well as the overlapping rate of the corresponding mask. In addition, to ensure the smoothness of the output displacement field, a regularization loss is introduced.

The similarity loss function between the shape-deformed image and the reference image is (Equation 10):

(10) $$L_{sim}(y_{i},x_{warp})=1-NCC(y_{i},x_{warp})=1-NCC(y_{i},\phi \circ x_{i})$$

Among them, the normalized cross-correlation (NCC) (39) function is used to measure the local similarity between two images, and its value ranges from 0 to 1, with higher values indicating stronger anatomical consistency. We specifically employ $1-NCC(y_{i},x_{warp})$ to quantify the similarity loss between follow-up images and their warped counterparts generated through deformable registration.

In addition, this study also introduces a regularization loss to constrain the similarity of the input image $x_{i}$ to the time interval $t_{itv}=0$, which is defined as (Equation 11):

(11) $$L_{reg}(x_{i},\phi_{0}\circ x_{i})=1-NCC(x_{i},\phi_{0}\circ x_{i})$$

$L_{reg}$ is used to regularize the follow-up time interval, that is, the predicted image at the time of baseline ( $t_{itv}=0$) should be consistent with the input image.

The deformation loss of the lung nodule mask adopts Tversky loss (42), which can alleviate the problem of imbalance between positive and negative samples. It is defined as (Equation 12):

(12) $$L_{mask}(s_{t},\phi \circ s_{i};\alpha ,\beta )=\frac{s_{t}\cap \phi \circ s_{i}}{s_{t}\cap \phi \circ s_{i}+\alpha |\phi \circ s_{i}-s_{t}|+\beta |s_{t}-\phi \circ s_{i}|}$$

Among them, α and β are used to control the direct balance of false positives and false negatives respectively. If we want to get a higher recall rate (how many samples are predicted to be positive among the actual positive samples), we can increase β to fulfill.

To encourage the smoothness of the displacement field u produced by the deformation prediction task, this study encourages a smooth output u by using a regularization of the gradient of the displacement field u, which is defined as (Equation 13):

(13) $$L_{smooth}(u)={\sum_{p\in \Omega }∥\nabla u(p)∥}^{2}$$

The total loss of the deformation prediction task branch can be described as (Equation 14):

(14) $$L_{warp}(x_{i},s_{i},u,y_{i})=L_{sim}(y_{i},\phi \circ x_{i})+\lambda_{1}L_{mask}(s_{t},\phi \circ s_{i})+\lambda_{2}L_{smooth}(u)+\lambda_{3}L_{reg}(\phi_{0})$$

where ${\text{λ}}_{\text{i}}$ represents the weight coefficient of each loss.

The texture prediction task needs to complete the texture prediction within the nodule voxel range based on the deformation task, so it measures the similarity between the texture prediction based on the input image ${\text{x}}_{\text{i}}$ and the reference image.

For the same follow-up nodule in the same patient, the images before and after scanning have certain similarities. This study uses L1 loss to improve the effect of texture prediction. The definition of L1 loss for (Equation 15):

(15) $$L_{sim\_}(\hat{y},y)=\frac{1}{\Omega }\sum_{p\in \Omega }\left|y-\hat{y}\right|$$

Similar to the deformation prediction task, the texture prediction task also includes a regularization loss function (Equation 16):

(16) $$L_{reg\_}(x_{res\_0})=\frac{1}{\Omega }{\sum_{p\in \Omega }\left|x_{res\_0}(p)\right|}^{2}$$

The total loss for the texture prediction task is (Equation 17):

(17) $$L_{texture}(\hat{y},y,x_{res\_0})=L_{sim}(\hat{y},y)+\lambda_{1}^{'}L_{reg\_}(x_{res\_0})$$

In addition, in the deformation prediction task, we use Tversky loss to better accommodate nodular regions, which may lead to more false-positive regions in the masked regions after deformation. Therefore, for the final evaluation of the lung nodule mask, we implemented the lung nodule segmentation task. In this task, we optimized the segmentation using the dice loss (43).

## 3 Results and discussion

### 3.1 Implementation details

The experimental platform used in this study is an Intel i7–8700 CPU with 32GB of RAM and one NVIDIA RTX 2080TI GPU with 11GB of memory. To further assess practical inference efficiency, the model was also evaluated on a mid-range configuration (Intel i5–10400 CPU, NVIDIA RTX 3060 12GB GPU), achieving an average processing speed of 4 nodules per second. The code for this paper is based on Python 3.6.13 and PyTorch 1.7.1 (44). In addition, MT-NoGNet uses AdamW (45) as the parameter optimizer. The learning rate for the shape prediction task is set to 0.001 (higher to accelerate convergence for global volumetric change learning) and 0.0005 for the texture prediction task (lower to ensure fine-grained density feature refinement), with a batch size of 8 balanced by hardware memory constraints and training stability. The weight coefficients of the loss functions for the shape prediction task are λ_1_ = 0.5, λ_2_ = 10, λ_3_ = 1: λ_2_ is assigned the highest weight to prioritize displacement field smoothness (consistent with physiological progressive nodule growth), λ_1_ balances mask overlap accuracy without over-constraining, and λ_3_ ensures temporal consistency at baseline (t_itv_=0). The Tversky loss coefficients are α=0.2 and β=0.8, selected to minimize false negatives (critical for clinical growth detection) while controlling excessive false positives, validated via pre-experiments comparing α/β combinations (0.1/0.9, 0.2/0.8, 0.3/0.7). The weight coefficient of the loss function for the texture prediction task is λ_1_^’^=1. Our sensitivity analysis confirmed that the model’s performance (measured by PSNR*) exhibited a fluctuation of less than 0.3 dB when key hyperparameters (e.g., λ_2_, β, learning rates) varied within ±20% of their set values, indicating robust parameter selection not overly tuned to the specific dataset. MT-NoGNet training includes two stages. In the first stage, we trained the deformation prediction task, texture prediction task and segmentation task simultaneously for a total of 200 epochs. In the second stage, we froze the shared encoder and only trained the decoder for each task separately, with 200 epochs per task.

### 3.2 Evaluation criteria

In order to evaluate the similarity between the nodule images obtained from the nodule growth prediction model and the follow-up images, this study used peak signal to noise ratio (PSNR), structural similarity index (SSIM), and dice similarity coefficient (DSC) to evaluate the quality of the predicted images and the nodule regions respectively.

The definition of PSNR is (Equation 18):

(18) $$PSNR=10\times \log_{10}(\frac{MAX_{img}^{2}}{\sqrt{MSE}})=20\times \log_{10}(\frac{MAX_{img}}{\sqrt{MSE}})$$

PSNR is used to measure the signal-to-noise ratio between two images, where $MAX_{img}$ is the maximum value of the pixel range of the image, and MSE is the mean square error between the two compared images.

Meanwhile, SSIM measures the similarity between two images from the aspects of brightness, contrast, and structure respectively. The definition of brightness similarity l(x,y) between two images is (Equation 19):

(19) $$l(x,y)=\frac{2\mu_{x}\mu_{y}+c_{1}}{\mu_{x}^{2}+\mu_{y}^{2}+c_{1}}$$

Among them, $\mu_{x}$ and $\mu_{y}$ represent the average value of two images respectively, and $c_{1}$ is a predefined constant.

The contrast similarity c(x,y) between two images is defined as (Equation 20):

(20) $$c(x,y)=\frac{2\sigma_{x}\sigma_{y}+c_{2}}{\sigma_{x}^{2}+\sigma_{y}^{2}+c_{2}}$$

Among them, $\sigma_{x}$ and $\sigma_{y}$ represent the variance of the two images respectively, and $c_{2}$ is a predefined constant.

The structural similarity s(x,y) between two images is defined as (Equation 21):

(21) $$s(x,y)=\frac{\sigma_{xy}+c_{3}}{\sigma_{x}\sigma_{y}+c_{3}}$$

Among them, $\sigma_{x}$ and $\sigma_{y}$ represent the variance of the two images respectively, and $c_{3}$ is a predefined constant.

The final SSIM is defined as (Equation 22):

(22) $$SSIM(x,y)=l{\left(x,y\right)}^{\alpha }c{\left(x,y\right)}^{\beta }s{\left(x,y\right)}^{\gamma }$$

Among them, *α, β, γ* are the weight coefficients of brightness, contrast and structure respectively.

For simplicity, the above parameters are generally set as: α=β=γ=1, $c_{3}=c_{2}/2$, and this paper also follows this setting. Therefore, Equation 22 can be simplified as (Equation 23):

(23) $$SSIM(x,y)=\frac{\left(2\mu_{x}\mu_{y}+c_{1})(2\sigma_{xy}+c_{2}\right)}{\left(\mu_{x}^{2}+\mu_{y}^{2}+c_{1})(\sigma_{x}^{2}+\sigma_{y}^{2}+c_{2}\right)}$$

DSC is defined as (Equation 24):

(24) $$DSC=\frac{2Gt\cap Seg}{Gt\cup Seg}$$

where *Gt* represents the result marked by the radiologist, and *Seg* represents the segmentation result of the model.

### 3.3 Overall performance evaluation

For the growth prediction of pulmonary nodules, this study used 5-fold cross-validation to evaluate the model given in this paper. Nodules are viewed as separate entities in order to partition the data. The experimental results of directly using U-Net to predict growth and the method proposed by Y Li et al. (31) were compared. This study also evaluated their method on the dataset proposed in this study. The comparison results are shown in **Table 1**. This study evaluated the model’s prediction performance on the entire input image and the nodule region separately. The nodule region only evaluated the prediction performance of the nodule part, not the irrelevant background region. It can be seen that the multitask model proposed in this study is significantly better than U-Net and the method in the literature (31) for predicting pulmonary nodule growth. It is worth noting that U-Net has better prediction performance on the overall image, but it has poor prediction performance on the nodule region, which indicates that the model is largely optimized for predicting the background rather than the main purpose of this study. In addition, the results show that adding the TEM module can improve the model’s prediction performance. The pulmonary nodule growth prediction framework proposed by Y Li et al. (31) uses two cascaded networks for deformation prediction and texture prediction, which not only increases the model’s parameters but also cannot use the features of the deformation prediction task and texture prediction task at the same time. The pulmonary nodule growth prediction method proposed in this study is better than their method in all indicators, and the PSNR index in the nodule region has improved by 0.32, and the SSIM index in the nodule region has improved by 0.0129.

**Table 1:** The prediction results of pulmonary nodule growth by different models.

Method	PSNR	PSNR*	SSIM	SSIM*	DSC
(Baseline)U-Net	19.30	43.31	0.5141	0.7034	–
U-Net+TEM	19.30	43.54	0.5176	0.7184	–
NoFoNet (31)	18.21	43.64	0.4887	0.7647	0.7692
Ours	18.22	44.30	0.4898	0.7776	0.7823

* represents the performance of nodule region.

This model’s differentiated performance between nodule-specific (PSNR, SSIM) and whole-image (PSNR, SSIM) metrics directly reflects its clinical task-oriented design. Baseline models like U-Net employ mean squared error loss functions that treat all voxels equally, resulting in excessive optimization of the predominant background regions while underemphasizing the critical evolution of internal nodule features. In contrast, our MT-NoGNet incorporates explicit supervision through nodule masks and utilizes mask-based texture fusion to concentrate the model’s learning capacity on the nodule region itself. This deliberate “focus shift” slightly reduces the fidelity of background reconstruction but substantially enhances the accuracy of predicting nodule volume and internal texture variations—such as density changes—which serve as key clinical indicators for malignancy risk assessment under guidelines like the Fleischner Society recommendations. Consequently, improvements in nodule-specific metrics carry greater clinical significance than the marginal declines observed in global image metrics.

### 3.4 Ablation experiment

In order to analyze the effectiveness of each module in this method, this study conducted ablation experiments to evaluate the impact of each module on experimental performance. The specific experimental results are shown in **Table 2**. Among them, WarpNet + TextureNet adopts the cascade of deformation prediction and texture prediction for pulmonary nodule growth prediction, which is a single-task mode. MT-NoGNet (base) only uses a multi-task structure, corresponding to a mean square error loss. The experimental results in **Table 2** show that pulmonary nodule growth prediction based on multi-task learning is more comprehensive than the cascade method. MT-NoGNet (base)+DS adds the proposed deep supervision (DS) module based on MT-NoGNet (base), and after adding this module, the PSNR and SSIM indicators in the nodule region of the model are improved, especially the SSIM indicator in the nodule region. MT-NoGNet + DS + TL further adds Tversky loss (TL) to the deformation prediction task. Although compared with MT-NoGNet + DS with only TL loss, MT-NoGNet + DS + TL performs better in SSIM indicators in the nodule region, while the addition of TL is better in the PSNR indicator in the nodule region. In addition, the purpose of the deformation prediction task is to determine the size of the region of interest of the nodule in the growth stage. The addition of TL can adjust the balance between false positives and false negatives in the mask region. The TL loss can relax the prediction loss of false positives and false negatives by adjusting their weights, allowing the deformation prediction task to have a certain degree of false positives to improve the effectiveness of the texture prediction task. This can alleviate the situation where too many false negatives occur in the deformation prediction stage, which leads to inaccurate predictions of nodule texture changes in the texture prediction task. This was confirmed in the configuration of MT-NoGNet + DS + TL + L1, which means that MT-NoGNet adds the deep supervision strategy TL loss and L1 loss, and the model’s SSIM and PSNR indicators in the nodule region are further improved after adding L1 loss, fully verifying the necessity of adding TL loss.

**Table 2:** Results of ablation experiments.

Method	PSNR	PSNR*	SSIM	SSIM*
WarpNet + TextureNet	18.21	43.66	0.4891	0.7638
MT-NoGNet (base)	18.22	43.92	0.4893	0.7688
MT-NoGNet + DS	18.22	44.13	0.4894	0.7742
MT-NoGNet + DS + TL	18.22	44.26	0.4897	0.7727
MT-NoGNet + DS + L1	18.22	44.15	0.4895	0.7757
MT-NoGNet + DS + TL + L1	18.22	44.30	0.4898	0.7776

### 3.5 Visual analysis

**Figure 3:**
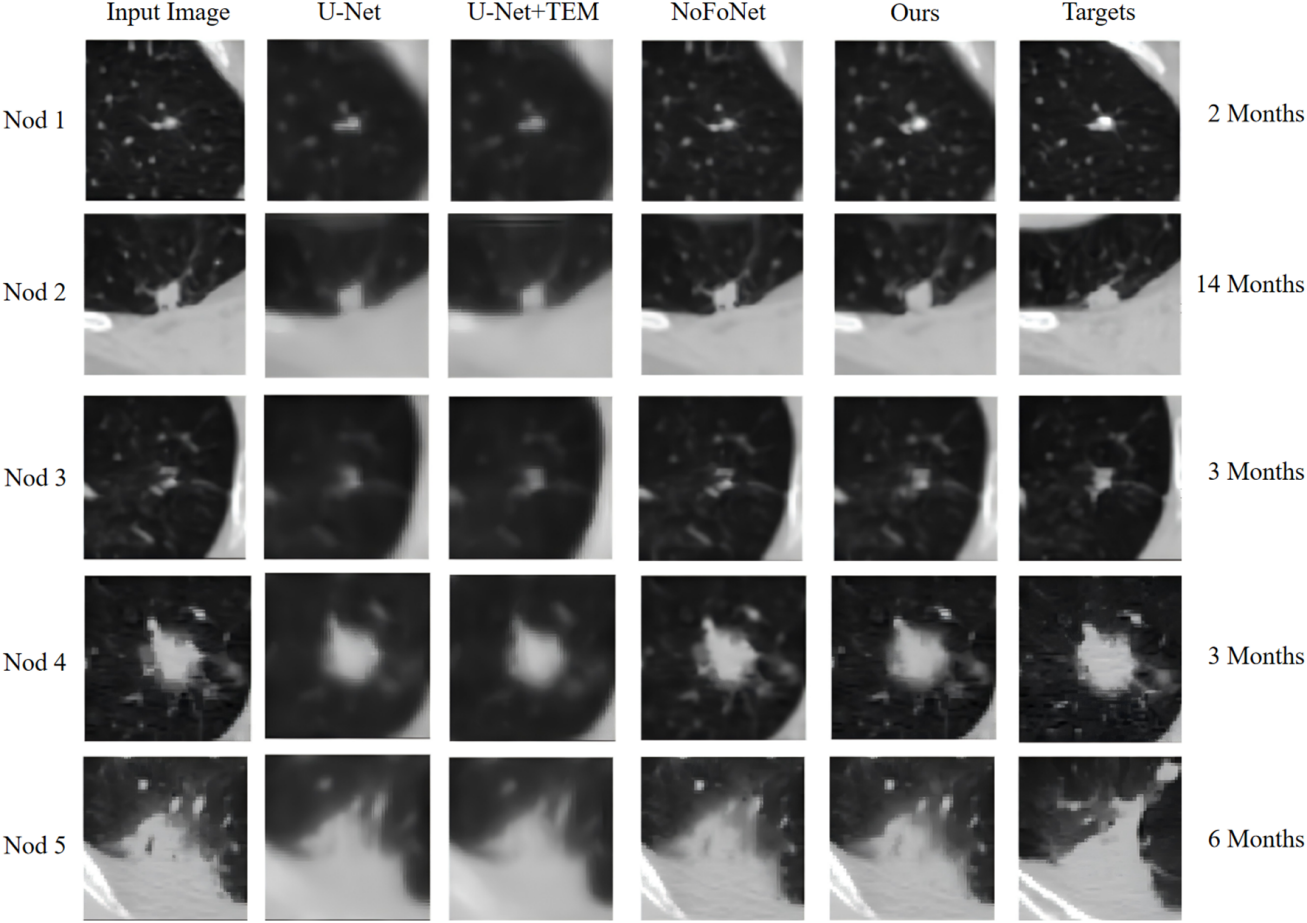
Comparison of the prediction effect of different models on the growth of pulmonary nodules.

**Figure 4:**
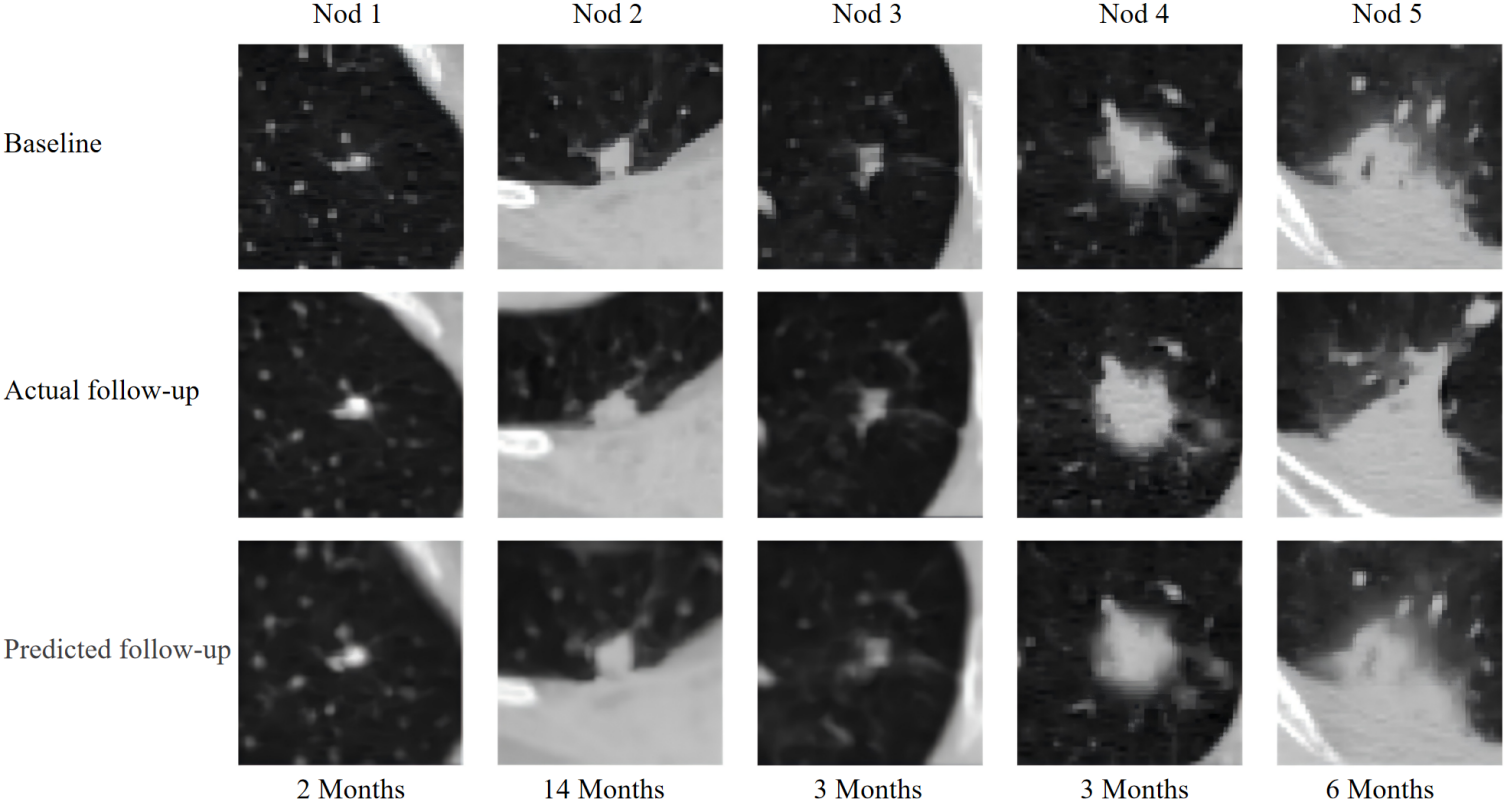
Nodule growth prediction examples. (Top) Baseline, (Middle) Actual follow-up, (Bottom) Predicted follow-up.

To further analyze the differences in the predictive performance of the proposed pulmonary nodule growth prediction model and other models, we demonstrate the effectiveness of the lung nodule methods proposed in this study and other methods for pulmonary nodule growth prediction, as shown in **Figure 3**. The second and third columns in **Figure 3** show the predictive performance of the U-Net and U-Net combined with the Time Encoding Module (TEM) models for pulmonary nodule growth images. Overall, both models cannot effectively predict the texture information of nodules, and the U-Net model with the TEM module is slightly better than the U-Net model alone, especially for nodule 3, where the predicted nodule is clearer in texture detail. This is because the growth of nodules is complex, and directly predicting the growth image of nodules is a very difficult problem. For the NoFoNet model proposed in reference (31), the model uses a strategy of deformation prediction and cascaded texture prediction, which has a significant performance improvement compared to U-Net type models and can clearly observe the texture changes of nodules, indicating that decomposing pulmonary nodule growth prediction into deformation prediction and texture prediction is effective. The fifth column in **Figure 3** shows that the pulmonary nodule growth prediction method proposed in this study can predict more realistic pulmonary nodule growth images compared to NoFoNet. For nodule 1, NoFoNet has a large difference in texture from the actual growth image of the nodule, and even some texture details are lost (the nodule is more blurry), while the method proposed in this paper can predict the density changes of the nodule during growth (the density of nodule 1 increases and appears bright in the image). In addition, for nodule 2, the proposed multi-task learning method predicts that the nodule will increase in volume, but NoFoNet predicts that the nodule will not undergo significant volume changes, indicating that the proposed multi-task learning pulmonary nodule growth prediction model can better learn the feature information of nodules. Accurate prediction of such nodules is crucial because nodules with increased volume are likely to develop into malignant nodules. For nodule 3, NoFoNet predicts that the nodule density is lower than the actual growth image, while the method proposed in this paper predicts that the density of the central region of the nodule is closer to the actual growth image, but some ground glass shadows outside the edge of the actual image that do not belong to the nodule are predicted by the proposed method, which may be due to the use of the TL loss in this paper, which expands the area of deformation prediction. For nodule 4, the method proposed in this paper predicts a result that is closer to the actual image, and NoFoNet also loses some of the nodule texture information. For nodule 5, the images predicted by the proposed method and NoFoNet are similar, both predicting some changes in detail, but there are still some differences from the actual growth image. The visualizations of the Baseline, Actual follow-up, and the Predicted follow-up from our model are detailed in **Figure 4**.

### 3.6 Clinical applicability analysis

Our quantitative improvements, particularly within the nodule region (PSNR, SSIM), suggest potential clinical relevance. By generating predictions of a nodule’s density and texture evolution—which correspond to key criteria in guidelines (8, 9)—the model provides a visual tool that may aid in the assessment of changes that are not fully captured by volume measurements alone. For instance, **Figure 3** shows our model correctly predicting density increase in Nodule 1. While the Dice score of 0.7823 can be improved, it already surpasses the clinical benchmark of 0.76 used by tools like ITK-SNAP. Our multi-task framework (DSC: 0.7823) also outperforms single-task segmentation (DSC: 0.7611). The model’s ability to output a synthesized follow-up image and mask supports its exploration for several clinical tasks, such as: providing a visual reference for assessing indeterminate nodules, offering a consistent automated measurement to complement growth analysis, and highlighting textural changes alongside dimensional ones.

To address the practical deployment feasibility, we evaluated the model’s computational efficiency and clinical integration pathway. The trained model demonstrates efficient inference capability, processing a single nodule volume in approximately 250 milliseconds on a mid-range GPU (NVIDIA RTX 3060), equivalent to 4 nodules per second, with memory consumption under 1.5 GB - well within the capacity of standard clinical workstations.

For seamless clinical adoption, the prediction outputs can be transformed into actionable decision support through three key mechanisms: (1) Visual Comparison Interface providing side-by-side display of baseline, predicted follow-up, and actual follow-up scans; (2) Structured Reporting automatically generating quantitative metrics (volume change rate, density evolution) alongside morphological descriptors; (3) Risk Stratification Alerts triggered when predicted features meet progression criteria in clinical guidelines like Lung-RADS. Such an integrated approach is designed to allow radiologists to evaluate model predictions within the existing workflow, thereby exploring the potential utility of growth prediction in nodule management.

### 3.7 Error analysis and robustness discussion

To address the reviewer’s comments and thoroughly evaluate the robustness of our model, we conducted a systematic qualitative error analysis by examining cases with the best and worst prediction performance (top and bottom 10% in DSC and PSNR) in conjunction with a radiologist. This analysis revealed that the model’s performance was predominantly influenced by the complexity of the nodule’s anatomical environment rather than by its density type. Specifically, poor predictions were frequently associated with complex contexts such as nodules adjacent to the pleura or accompanied by severe fibrosis or vascular convergence, where the model struggled to differentiate genuine nodule growth from structural changes in surrounding tissues. In contrast, predictions were most accurate for well-circumscribed, isolated nodules with regular morphology. Furthermore, while a formal subgroup analysis was not performed due to sample size constraints, a holistic review of all test cases indicated no clear correlation between model performance and initial nodule volume or volume change rate. Prediction successes and failures were observed across all size categories, with the primary cause of inaccuracy consistently linked to anatomical complexity. These observations suggest that MT-NoGNet exhibits potential robustness across nodules of different sizes, with its performance boundary being defined more by anatomical context than by intrinsic nodule characteristics such as size or density type.

### 3.8 Multi-task output consistency analysis

To further validate the consistency and reliability of the proposed multi-task model in predicting nodule structure and texture, we conducted a validation experiment in which an independent pre-trained 3D U-Net segmentation model was used to segment the generated nodule growth prediction images, yielding “secondary segmentation masks.” These were then compared with the segmentation results directly output by the proposed model. This experiment aimed to evaluate whether the model maintains anatomical consistency during multi-task learning, thereby enhancing the credibility of its outputs.

All predicted nodule images from the test set (246 samples in total) were analyzed using the pre-trained 3D U-Net model. The Dice Similarity Coefficient (DSC) was calculated between the secondary segmentation masks and the ground truth masks annotated by radiologists in the actual follow-up images. The results showed that the average DSC of the secondary segmentations was 0.812 ± 0.108 (range: 0.0095–0.9345), with approximately 81.2% of samples achieving a DSC above 0.75. This indicates that the vast majority of the predicted images retain structurally plausible characteristics amenable to segmentation. Overall, the model demonstrates maintained anatomical rationality across multi-task outputs, providing a reliable structural foundation for subsequent automated analysis and diagnosis in clinical applications.

## 4 Conclusions

In this study, we propose a synergistic multi-task learning framework for pulmonary nodule growth prediction, integrating three complementary diagnostic dimensions: anatomical segmentation, deformation mapping, and texture evolution analysis. Our architecture achieves effective feature fusion through three key innovations: a parameter-shared encoder that establishes cross-task correlations, a progressive supervision mechanism combining direct feature guidance with residual-constrained optimization, and multi-scale feature recalibration modules that enhance the model’s ability to capture longitudinal changes. The main contribution of our framework lies in its synergistic prediction of clinically relevant features—density, texture, and morphology. While the absolute performance improvements may seem incremental, they have a direct clinical impact by aiding in the identification of risk factors specified in current clinical guidelines, providing a solid foundation for the integration of deep learning techniques in precise nodule management.

Both qualitative and quantitative results underscore the model’s potential in generating growth projections that are interpretable to radiologists, particularly in identifying subtle texture transitions that precede volumetric changes. However, the present study has several limitations. The model’s performance may be constrained when applied to nodules with unusually long follow-up intervals, which exceed the range of our dataset, or to those showing atypical, rapid morphological progression. These cases were underrepresented in our training cohort, highlighting the model’s reliance on the existing data distribution and suggesting that its generalizability to such edge cases requires further validation.

Moreover, the model was developed using a single-center retrospective dataset, which could result in performance biases influenced by the specific imaging protocols and patient populations of this center. Thus, the generalizability of the model to different scanning devices or patient demographics remains uncertain and should be further evaluated through multi-center external validation. Additionally, the dataset may have lacked sufficient coverage of certain complex nodule types, such as those adjacent to the pleura or associated with severe fibrosis. This limitation is consistent with the observed variability in model performance in complex anatomical contexts, as noted in our error analysis. Finally, the current evaluation relies primarily on image similarity metrics, and the direct correlation between the model’s predictions and clinical outcomes—such as pathological diagnosis or patient prognosis—requires validation through prospective clinical studies. Future work will therefore focus on multi-center validation, prospective assessment of clinical utility, and the development of more advanced spatiotemporal growth dynamics models to better capture the non-linear growth patterns of pulmonary nodules. These efforts aim to address the identified limitations and further bridge the gap between technical development and clinical application in pulmonary nodule follow-up.

## Data Availability

The original contributions presented in the study are included in the article/supplementary material. Further inquiries can be directed to the corresponding author/s.

## References

[1] Pelc NJ . Recent and future directions in CT imaging. Ann BioMed Eng. (2014) 42:260–8. doi: 10.1007/s10439-014-0974-z, PMID: 24435658 PMC3958932

[2] Heuvelmans MA Oudkerk M de Bock GH de Koning HJ Xie X van Ooijen PM . Optimisation of volume-doubling time cutoff for fast-growing lung nodules in CT lung cancer screening reduces false-positive referrals. Eur Radiol. (2013) 23:1836–45. doi: 10.1007/s00330-013-2799-9, PMID: 23508275

[3] Larici AR Farchione A Franchi P Ciliberto M Cicchetti G Calandriello L . Lung nodules: size still matters. Eur Respir Rev. (2017) 26:170025. doi: 10.1183/16000617.0025-2017, PMID: 29263171 PMC9488618

[4] Ko JP Berman EJ Kaur M Babb JS Bomsztyk E Greenberg AK . Pulmonary Nodules: growth rate assessment in patients by using serial CT and three-dimensional volumetry. Radiology. (2012) 262:662–71. doi: 10.1148/radiol.11100878, PMID: 22156993 PMC3267080

[5] Liao F Liang M Li Z Hu X Song S . Evaluate the Malignancy of pulmonary nodules using the 3-D deep leaky noisy-OR network. IEEE Trans Neural Netw Learn Syst. (2019) 30:3484–95. doi: 10.1109/TNNLS.2019.2892409, PMID: 30794190

[6] Zhu WT Liu CC Fan W Xie XH . DeepLung: deep 3D dual path nets for automated pulmonary nodule detection and classification. IEEE Wint Conf Appl. (2018) 673–81. doi: 10.1109/WACV.2018.00079

[7] Hwang EJ Goo JM Kim HY Yi J Kim Y . Optimum diameter threshold for lung nodules at baseline lung cancer screening with low-dose chest CT: exploration of results from the Korean Lung Cancer Screening Project. Eur Radiol. (2021) 31:7202–12. doi: 10.1007/s00330-021-07827-8, PMID: 33738597

[8] Wood DE . National comprehensive cancer network (NCCN) clinical practice guidelines for lung cancer screening. Thorac Surg Clin. (2015) 25:185–97. doi: 10.1016/j.thorsurg.2014.12.003, PMID: 25901562

[9] MacMahon H Naidich DP Goo JM Lee KS Leung ANC Mayo JR . Guidelines for management of incidental pulmonary nodules detected on CT images: from the fleischner society 2017. Radiology. (2017) 284:228–43. doi: 10.1148/radiol.2017161659, PMID: 28240562

[10] Pinsky PF Gierada DS Black W Munden R Nath H Aberle D . Performance of Lung-RADS in the National Lung Screening Trial: a retrospective assessment. Ann Intern Med. (2015) 162:485–91. doi: 10.7326/M14-2086, PMID: 25664444 PMC4705835

[11] Setio AA Ciompi F Litjens G Gerke P Jacobs C van Riel SJ . Pulmonary nodule detection in CT images: false positive reduction using multi-view convolutional networks. IEEE Trans Med Imaging. (2016) 35:1160–9. doi: 10.1109/TMI.2016.2536809, PMID: 26955024

[12] Alakwaa W Nassef M Badr A . Lung cancer detection and classification with 3D convolutional neural network (3D-CNN). Int J Adv Comput Sc. (2017) 8:410–7. doi: 10.14569/IJACSA.2017.080853

[13] Dou Q Chen H Jin Y Lin H Qin J Heng P-A . (2017). Automated pulmonary nodule detection via 3d convnets with online sample filtering and hybrid-loss residual learning, in: Medical Image Computing and Computer Assisted Intervention– MICCAI 2017: 20th International Conference, Quebec City, QC, Canada, September 11-13, 2017. (Canada: Springer Verlag) pp. 630–8.

[14] Wang J Wang JW Wens YF Lu HB Niu TY Pan JF . Pulmonary nodule detection in volumetric chest CT scans using CNNs-based nodule-size-adaptive detection and classification. IEEE Access. (2019) 7:46033–44. doi: 10.1109/ACCESS.2019.2908195

[15] Ozdemir O Russell RL Berlin AA . A 3D probabilistic deep learning system for detection and diagnosis of lung cancer using low-dose CT scans. IEEE Trans Med Imaging. (2020) 39:1419–29. doi: 10.1109/TMI.2019.2947595, PMID: 31675322

[16] Tajbakhsh N Suzuki K . Comparing two classes of end-to-end machine-learning models in lung nodule detection and classification: MTANNs vs. CNNs. Pattern Recogn. (2017) 63:476–86. doi: 10.1016/j.patcog.2016.09.029

[17] Jiang HY Ma H Qian W Gao MD Li Y . An automatic detection system of lung nodule based on multigroup patch-based deep learning network. IEEE J BioMed Health. (2018) 22:1227–37. doi: 10.1109/JBHI.2017.2725903, PMID: 28715341

[18] Luo XD Song T Wang GT Chen JN Chen YA Li K . SCPM-Net: An anchor-free 3D lung nodule detection network using sphere representation and center points matching. Med Image Anal. (2022) 75:102295-301. doi: 10.1016/j.media.2021.102287, PMID: 34731775

[19] Wang S Zhou M Liu Z Liu Z Gu D Zang Y . Central focused convolutional neural networks: Developing a data-driven model for lung nodule segmentation. Med Image Anal. (2017) 40:172–83. doi: 10.1016/j.media.2017.06.014, PMID: 28688283 PMC5661888

[20] Liu H Cao H Song E Ma G Xu X Jin R . A cascaded dual-pathway residual network for lung nodule segmentation in CT images. Phys Med. (2019) 63:112–21. doi: 10.1016/j.ejmp.2019.06.003, PMID: 31221402

[21] Wu ZT Zhou QJ Wang F . Coarse-to-fine lung nodule segmentation in CT images with image enhancement and dual-branch network. IEEE Access. (2021) 9:7255–62. doi: 10.1109/ACCESS.2021.3049379

[22] Cao HC Liu H Song EM Hung CC Ma GZ Xu XY . Dual-branch residual network for lung nodule segmentation. Appl Soft Comput. (2020) 86:105934. doi: 10.1016/j.asoc.2019.105934

[23] Soares AR Lima TJ Ricardo de Andrade LR Rodrigues JJ Araujo FH . (2021). Automatic segmentation of lung nodules in CT images using deep learning, in: 2020 IEEE International Conference on E-health Networking, Application & Services (HEALTHCOM). [Shenzhen, China: Institute of Electrical and Electronics Engineers Inc. (IEEE)]. pp. 1–6.

[24] Wu WH Gao L Duan HH Huang G Ye XD Nie SD . Segmentation of pulmonary nodules in CT images based on 3D-UNET combined with three-dimensional conditional random field optimization. Med Phys. (2020) 47:4054–63. doi: 10.1002/mp.14248, PMID: 32428969

[25] Liu XL Hou F Qin H Hao AM . Multi-view multi-scale CNNs for lung nodule type classification from CT images. Pattern Recogn. (2018) 77:262–75. doi: 10.1016/j.patcog.2017.12.022

[26] Shen W Zhou M Yang F Yu DD Dong D Yang CY . Multi-crop Convolutional Neural Networks for lung nodule Malignancy suspiciousness classification. Pattern Recogn. (2017) 61:663–73. doi: 10.1016/j.patcog.2016.05.029

[27] Xu XY Wang CD Guo JX Gan YC Wang JY Bai HL . MSCS-DeepLN: Evaluating lung nodule Malignancy using multi-scale cost-sensitive neural networks. Med Image Anal. (2020) 65:101772. doi: 10.1016/j.media.2020.101772, PMID: 32674041

[28] Ardila D Kiraly AP Bharadwaj S Choi B Reicher JJ Peng L . End-to-end lung cancer screening with three-dimensional deep learning on low-dose chest computed tomography. Nat Med. (2019) 25:1319–9. doi: 10.1038/s41591-019-0536-x, PMID: 31253948

[29] Huang P Lin CT Li YL Tammemagi MC Brock MV Atkar-Khattra S . Prediction of lung cancer risk at follow-up screening with low-dose CT: a training and validation study of a deep learning method. Lancet Digit Health. (2019) 1:E353–62. doi: 10.1016/S2589-7500(19)30159-1, PMID: 32864596 PMC7450858

[30] Rafael-Palou X Aubanell A Bonavita I Ceresa M Piella G Ribas V . Re-Identification and growth detection of pulmonary nodules without image registration using 3D siamese neural networks. Med Image Anal. (2021) 67:101823. doi: 10.1016/j.media.2020.101823, PMID: 33075637

[31] Li Y Yang J Xu Y Xu J Ye X Tao G . (2020). Learning tumor growth via follow-up volume prediction for lung nodules, in: Medical Image Computing and Computer Assisted Intervention–MICCAI 2020: 23rd International Conference, Lima, Peru, October 4–8, 2020. (Cham, Switzerland: Springer). pp. 508–17.

[32] Yan K Wang X Lu L Summers RM . DeepLesion: automated mining of large-scale lesion annotations and universal lesion detection with deep learning. J Med Imaging (Bellingham). (2018) 5:36501. doi: 10.1117/1.JMI.5.3.036501, PMID: 30035154 PMC6052252

[33] Wolterink JM Leiner T de Vos BD van Hamersvelt RW Viergever MA Išgum I . Automatic coronary artery calcium scoring in cardiac CT angiography using paired convolutional neural networks. Med Image Anal. (2016) 34:123–36. doi: 10.1016/j.media.2016.04.004, PMID: 27138584

[34] Xie Y Xia Y Zhang J Song Y Feng D Fulham M . Knowledge-based collaborative deep learning for benign-malignant lung nodule classification on chest CT. IEEE Trans Med Imaging. (2019) 38:991–1004. doi: 10.1109/TMI.2018.2876510, PMID: 30334786

[35] Liu LH Dou Q Chen H Qin J Heng PA . Multi-task deep model with margin ranking loss for lung nodule analysis. IEEE T Med Imaging. (2020) 39:718–28. doi: 10.1109/TMI.2019.2934577, PMID: 31403410

[36] Wang CL . Segmentation of multiple structures in chest radiographs using multi-task fully convolutional networks. Image Analysis Scia 2017. (2017) 10270:282–9. doi: 10.1007/978-3-319-59129-2_24

[37] Wu BT Zhou Z Wang JW Wang YZ . Joint learning for pulmonary nodule segmentation, attributes and Malignancy prediction. I S BioMed Imaging. (2018), 1109–13. doi: 10.1109/ISBI.2018.8363765

[38] Liu WH Liu XB Li HY Li MC Zhao XM Zhu Z . Integrating lung parenchyma segmentation and nodule detection with deep multi-task learning. IEEE J BioMed Health. (2021) 25:3073–81. doi: 10.1109/JBHI.2021.3053023, PMID: 33471772

[39] Balakrishnan G Zhao A Sabuncu MR Guttag J Dalca AV . VoxelMorph: A learning framework for deformable medical image registration. IEEE T Med Imaging. (2019) 38:1788–800. doi: 10.1109/TMI.2019.2897538, PMID: 30716034

[40] Isensee F Maier-Hein KH . An attempt at beating the 3D U-Net. arXiv preprint arXiv:1908.02182. (2019). doi: 10.24926/548719

[41] Zeng GD Yang X Li J Yu LQ Heng PA Zheng GY . 3D U-net with multi-level deep supervision: fully automatic segmentation of proximal femur in 3D MR images. Mach Learn Med Imaging (Mlmi 2017). (2017) 10541:274–82. doi: 10.1007/978-3-319-67389-9_32

[42] Salehi SSM Erdogmus D Gholipour A . Tversky loss function for image segmentation using 3D fully convolutional deep networks. Mach Learn Med Imaging (Mlmi 2017). (2017) 10541:379–87. doi: 10.48550/arXiv.1706.05721

[43] Milletari F Navab N Ahmadi SA . V-net: fully convolutional neural networks for volumetric medical image segmentation. Int Conf 3d Vision. (2016), 565–71. doi: 10.1109/3DV.2016.79

[44] Paszke A Gross S Massa F Lerer A Bradbury J Chanan G . PyTorch: an imperative style, high-performance deep learning library. Adv Neural Inf Process Syst 32 (NeurIPS 2019). (2019) 8024-35. doi: 10.48550/arXiv.1912.01703

[45] Loshchilov I Hutter F . Fixing weight decay regularization in adam. ArXiv abs/1711.05101. (2017). doi: 10.48550/arXiv.1711.05101

